# Compression of the Femoral Vessels by a Pseudotumor after Metal-on-Metal Total Hip Arthroplasty

**DOI:** 10.1155/2017/2594902

**Published:** 2017-10-01

**Authors:** Yasuaki Tamaki, Tomohiro Goto, Takahiko Tsutsui, Tomoya Takasago, Keizo Wada, Koichi Sairyo

**Affiliations:** ^1^Department of Orthopedics, Takamatsu Red Cross Hospital, 4-1-3 Ban-cho, Takamatsu, Kagawa 760-0017, Japan; ^2^Department of Orthopedics, Institute of Biomedical Sciences, Tokushima University Graduate School, 3-18-15 Kuramoto, Tokushima 770-8503, Japan

## Abstract

Here we present a case of pseudotumor following total hip arthroplasty (THA) that resulted in a circulatory disturbance caused by compression of the femoral vasculature. A 63-year-old man presented with pain, swelling, and redness of the left leg 5 years after primary metal-on-metal THA using the AML-Plus stem, Pinnacle® acetabular cup, and 36 mm diameter Ultamet™ metal head system (DePuy Orthopaedics, Warsaw, IN). Enhanced computed tomography and magnetic resonance imaging revealed a large cystic lesion extending from the left hip anteriorly to the intrapelvic region and compressing the left femoral vessels. Percutaneous puncture of the lesion yielded a dark red aspirate and the patient was diagnosed to have a pseudotumor causing compression of the femoral vessels. We performed revision surgery to replace the metal head and metal liner with a smaller ceramic head and polyethylene liner without removal of the stem. Corrosion of the head-neck junction was identified intraoperatively with no obvious wear on the bearing surfaces. The left leg swelling and redness improved immediately postoperatively. A large pseudotumor should be kept in mind as a cause of vascular compression with unilateral leg edema in a patient who has undergone metal-on-metal THA.

## 1. Introduction

Total hip arthroplasty (THA) is a common treatment option for osteoarthritis of the hip. Various artificial materials can be used for the joint surface in THA, including metal, ceramics, and polyethylene, as well as combinations of these materials, including metal-on-polyethylene, metal-on-metal (MoM), ceramic-on-ceramic, and ceramic-on-polyethylene. The original MoM bearings have been steadily reintroduced over the past 20 years because of lower volumetric wear rates in comparison with conventional metal-on-polyethylene bearings [1]. Furthermore, a MoM bearing surface has the potential to reduce wear-induced osteolysis and provide greater stability because of the option of using a larger metal head [1, 2]. However, the early 2000s have seen an increasing number of publications regarding pseudotumors associated with a MoM bearing surface and metal corrosion in MoM THA. A pseudotumor associated with THA is a large fluid-filled or solid tissue mass adjacent to the implant and is hypothesized to result in part from an inflammatory foreign body reaction to the metal ion particles released from the bearing surfaces or corrosion at the metal trunnion. The presence of a pseudotumor contributes to clinically relevant pain/discomfort, periprosthetic soft tissue damage, and functional limitations. This entity is known as an aseptic lymphocyte-dominated vasculitis-associated lesion (ALVAL) or adverse reaction to metal debris [3, 4]. According to the literature, the most common clinical symptom in patients with a pseudotumor is pain in the hip, groin, or thigh [5]. Other relatively minor symptoms, including instability, weakness, palpable mass, hip discomfort without flank pain, and a localized rash have also been reported [5, 6]. Here we present the case of a patient with a pseudotumor after MoM THA who developed a circulatory disturbance caused by compression of the femoral vasculature. The patient consented to his case being submitted for publication.

## 2. Case Presentation

**Figure 1:**
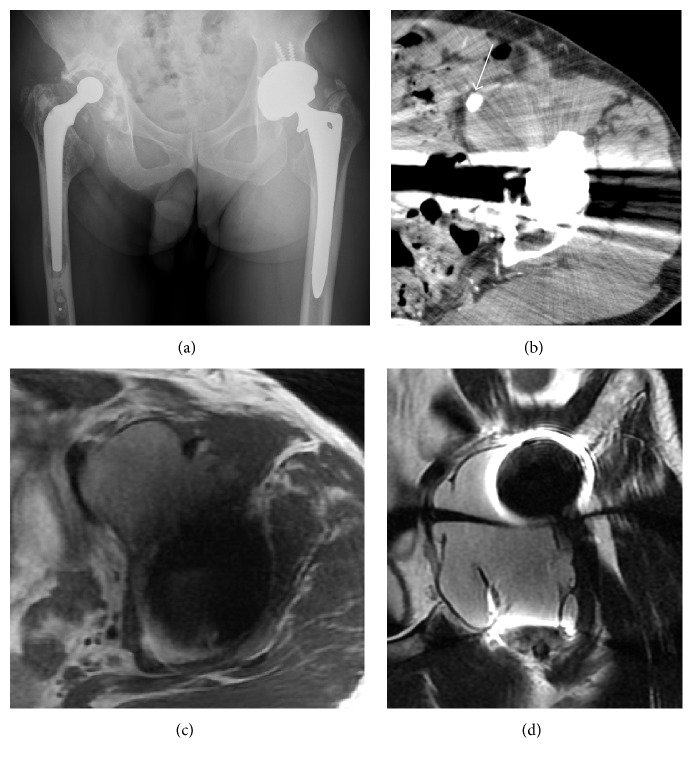
Plain radiographic, contrast-enhanced computed tomographic, and magnetic resonance images of the hip. Anteroposterior view on plain radiography shows no abnormal findings around the left hip (a). Axial view on contrast-enhanced computed tomography reveals a cystic lesion at the anterior aspect of the left hip compressing the left femoral vessels (white arrow, (b)). Magnetic resonance imaging reveals a cystic lesion extending from the anterior aspect of the left hip to the intrapelvic region. The mass has iso-high intensity on a T1-weighted axial image (c) and high intensity on a T2-weighted coronal image (d).

A 63-year-old man underwent MoM THA in another hospital with the AML-Plus stem, Pinnacle acetabular cup, and 36-mm diameter Ultamet metal head system (DePuy Orthopaedics, Warsaw, IN) for osteoarthritis of the left hip attributable to developmental dysplasia of the hip. His immediate postoperative course was uneventful. Five years later, he noticed slight pain in the left lower leg and thigh that gradually worsened. This progressed to acute swelling and redness on the left leg, so he visited the Department of Cardiovascular Medicine at our hospital. The attending physician suspected a deep vein thrombosis of the left leg and admitted the patient to hospital as an emergency. Venous ultrasonography of the left leg revealed no venous thrombosis but did show a cystic lesion extending along the iliopsoas tendon from the anterior aspect of the left hip to the intrapelvic region, so a referral was made to our department. At his initial consultation with us, the patient was 160 cm in height and weighed 60 kg (body mass index 23.4). On physical examination, the left thigh circumference was significantly increased compared with the contralateral side (42.2 cm versus 37.4 cm). Heat, redness, and swelling of the entire left leg were observed. There was no evidence of stem loosening, subsidence, or osteolysis around the stem and cup on plain radiography (Figure 1(a)). Contrast-enhanced computed tomography revealed a large cystic lesion extending from the anterior aspect of the left hip to the intrapelvic region and compressing the left femoral artery and vein (Figure 1(b)). Magnetic resonance imaging (MRI) also showed a well-defined cystic lesion (Figures 1(c) and 1(d)). An axial view on MRI at the largest part of the intrapelvic cystic lesion revealed a well-circumscribed, soft tissue lesion measuring 55.7 × 41.8 mm. The mass was slightly hyperintense to skeletal muscle at the center and very hypointense at the periphery of the lesion on T1-weighted imaging. On T2-weighted imaging, the mass showed high signal intensity at the center with low intensity at the rim. The mass extended distally along the iliopsoas tendon to the hip joint. According to the magnetic resonance grading system for pseudotumors [7], this patient was deemed to have moderate MoM disease. We performed percutaneous puncture of the cystic lesion under sonographic guidance and aspirated 30 mL of dark red fluid. There was no evidence of infection on bacterial culture of the aspirate and no laboratory findings suggestive of inflammation. Therefore, we made a diagnosis of pseudotumor associated with MoM THA.

**Figure 2:**
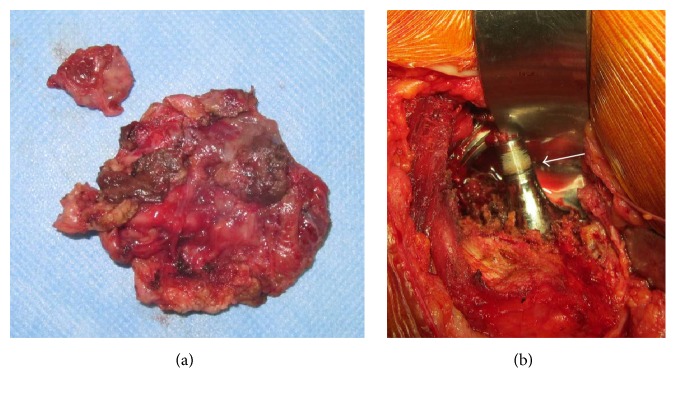
(a) Photograph of the resected pseudotumor. (b) Intraoperative photograph of the stem trunnion showing corrosion of the head-neck junction with black debris adhered to the trunnion (white arrow).

**Figure 3:**
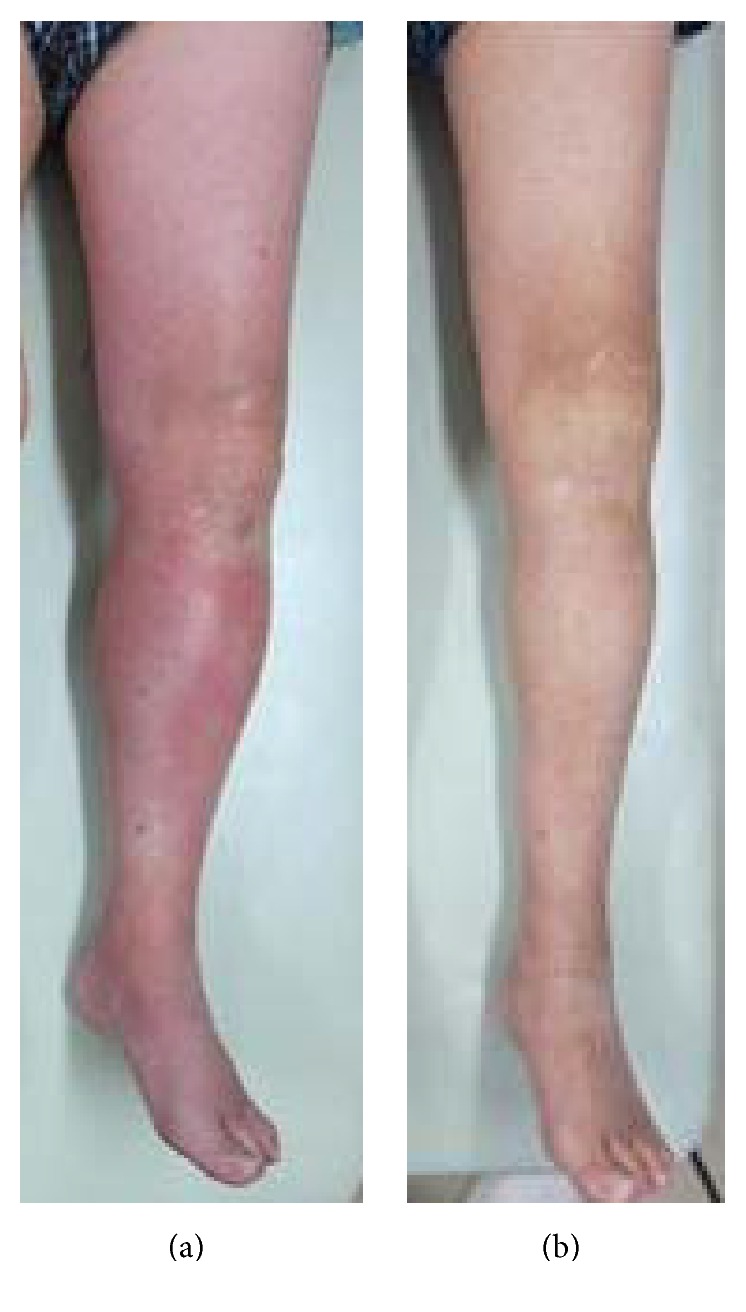
(a) Preoperative photograph showing left leg swelling and redness. (b) Photograph taken 3 weeks after revision surgery showing almost complete resolution of inflammatory changes in the left leg.

In a salvage revision procedure, we resected the pseudotumor and changed the metal head and liner. First, we made an incision via an ilioinguinal approach and resected the pseudotumor enveloped in the iliac muscle (Figure 2(a)). The resected pseudotumor was covered by a thick granulomatous capsule (approximately 1 cm thick) containing dark red fluid. We then replaced the metal liner with a polyethylene liner and the metal head with a 28-mm ceramic head (DePuy Orthopaedics) via a posterolateral approach. Intraoperatively, there was no obvious black metal debris inside the joint or the pseudotumor. There was also no evidence of abnormal friction between the metal head and liner. However, corrosion of the head-neck junction with black metal debris was detected around the trunnion (Figure 2(b)). Intraoperative bacterial culture was negative. Histologic analysis showed no cellular components inside the tumor but numerous foamy macrophages and a lymphocytic infiltrate at the periphery. No metal particles were evident. The left leg swelling and redness improved immediately after surgery and the inflammatory changes were almost resolved at 3 weeks postoperatively (Figure 3). At the most recent follow-up 3 years after surgery, the patient could walk unaided and was independent in activities of daily living, with no complications such as infection, dislocation of the hip, or recurrence of a pseudotumor.

## 3. Discussion

There is widespread concern regarding the incidence of ALVAL after MoM or metal-on-polyethylene THA. This pathologic entity has been described in various reports as a pseudotumor [6, 8, 9]. Bolland et al. reported an unacceptably high rate of failure in their MoM THA series, with evidence of abnormal wear at the trunnion-head interface and corrosion of the neck surface [10]. These failures might reasonably be thought to be the result of a mismatch between the head and stem taper/trunnion. In our case, there was no mismatch between the trunnion and head; the head and the trunnion both had a 9/10 taper. However, larger heads require higher assembly forces because the taper interface must be able to withstand the higher joint friction moments [2]. This force may generate micromotion at the head-neck junction. This was one of the causes of the trunnionosis in our patient, in whom we used a 36-mm diameter metal head. Moreover, the AML-Plus stem with a 36-mm (or larger) diameter head is suspected to be associated with a higher risk of pseudotumor associated with trunnionosis. The 9/10 taper trunnion of the AML-Plus stem is smaller than that of other commonly used implants, which have a 12/14 taper trunnion. A small taper translates to a smaller surface area, thereby increasing the potential for micromotion at the trunnion interface [11].

There is no consensus regarding how to manage revision surgery for a pseudotumor. We performed revision surgery by replacing the metal head and metal liner with a smaller ceramic head and polyethylene liner. An ideal method is to replace all components, including the stem, head, and liner. However, the AML-Plus stem is rigidly fixed because of its fully porous-coated surface, making it extremely difficult to remove. We performed revision surgery by replacing the 36-mm metal head with a 28-mm ceramic head. Changing to a smaller head decreases the oscillation angle of the hip joint. However, in our patient, the left hip joint showed contracture; the stability of the joint was good during surgery by slight leg lengthening. Therefore, we considered that a large head was not needed to stabilize the joint in this patient. We consider that a smaller head is a suitable option for revision surgery because it decreases the assembly forces at the trunnion. The manufacturer of the AML-Plus stem recommended replacement of the metal revision head implant. However, we could not exclude the possibility of recurrence of trunnionosis between the replaced metal head and the damaged neck, so we avoided this option. We also considered using a ceramic head with an internal titanium sleeve to protect the ceramic material but found that there was no titanium sleeve available for a 9/10 taper. Therefore, we decided to use a ceramic head for revision surgery after the potential risk of ceramic fracture by coupling with a damaged trunnion or recurrence of trunnionosis between the replaced metal head and the damaged neck had been explained to the patient and his family.

In a series reported by Kwon et al. [5], 70% of patients in whom a pseudotumor was detected on MRI were symptomatic and the most common symptom was pain at the hip, groin, or thigh. Instability, weakness, a palpable mass, hip discomfort without flank pain, and a localized rash were also detected. There have been several reports of patients in whom the femoral vessels became compressed by a large pseudotumor and whose presentation was similar to that of our patient [12, 13]. Basically, progression of osteolysis or a pseudotumor associated with a hip prosthesis tends to extend around the iliopsoas tendon or external rotator muscles because of the pressure gradient in the effective joint space; that is, a pseudocapsule generated after THA has some weak areas where herniation or extrusion into the joint space is more likely to occur [14]. Patients with a mass extending anteriorly along the iliopsoas tendon may be at increased risk of compression of the femoral vasculature. Compression of the femoral vein may cause venous stasis, edema, and femoral vein thrombosis. Femoral vein thrombosis is particularly worrisome because of its potential for a fatal outcome, such as pulmonary embolus.

In conclusion, we have encountered a case of a large pseudotumor that caused direct compression of the femoral vessels. The possibility of a large pseudotumor should be kept in mind as a cause of venous compression, particularly if it involves the anterior aspect of the hip or intrapelvic region. Early examination of the circulation is important when unilateral leg edema is observed in a patient who has undergone MoM THA.
